# Achieving Fast Charging and Superior Cycling Stability Single‐Crystal Ni‐Rich Cathodes by Ultrafast Aqueous Washing

**DOI:** 10.1002/advs.202517421

**Published:** 2025-12-23

**Authors:** Kaixin Liu, Jia Yang, Huaping Wang, Yongtao Tan, Dongdong Fan, Yongjian Cui, YunJian Liu, Xiaoyan Li, Hailong Wang

**Affiliations:** ^1^ School of Materials and New Energy Ningxia University Yinchuan China; ^2^ Helanshan Laboratory Yinchuan China; ^3^ School of Materials Science & Engineering Jiangsu University Zhenjiang China; ^4^ Ningxia Hanyao Rich Lithium Technology Co., Ltd. Yinchuan China

**Keywords:** cycling stability, fast charging capability, Ni‐rich oxides, single crystal

## Abstract

Single‐crystal Ni‐rich layered oxides represent ideal cathodes for high‐energy‐density lithium‐ion batteries due to their high compaction density and volumetric capacity. However, lithium‐excess high‐temperature synthesis generates substantial residual lithium compounds (RLCs; e.g., Li_2_CO_3_/LiOH) on particle surfaces. These RLCs elevate interfacial impedance, while protracted Li⁺ diffusion pathways in micrometer‐sized crystals collectively degrade fast‐charge capability and cycling stability. To address these challenges, we develop an efficient strategy employing 3‐min ultrafast washing followed by 600°C annealing for LiNi_0.85_Co_0.1_Mn_0.05_O_2_ single crystals. This approach achieves 80% capacity retention at 5 C charging meeting extreme fast‐charging targets while maintaining superior cycling stability at 1 C. The ultrafast wash removes 94.5% of surface RLCs while confining structural deterioration to a mere 2–4 nm rock‐salt reconstruction layer. Complementarily, in situ X‐ray diffraction (XRD) reveals that this attenuated surface rock‐salt phase enables smooth bulk phase transitions during Li^+^ (de)intercalation, thereby enhancing structural reversibility, ionic conductivity, and long‐term stability.

## Graphical abstract

Prolonged water washing severely damages the surface layered structure of single‐crystal Ni‐rich cathodes, forming a thick rock‐salt barrier. In contrast, our 3‐min ultrafast washing removes residual lithium while confining surface reconstruction to an ultrathin 2–4 nm layer, thereby unlocking excellent fast‐charging and cycling stability.

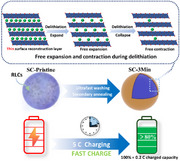

## 1 Introduction

Lithium‐ion batteries (LIBs), serving as the primary power source for electric vehicles, exhibit energy density, and cycle life critically dependent on the structural stability of cathode materials [1]. Ni‐rich layered oxide cathodes (LiNi_x_Co_y_Mn_z_O_2_, x ≥0.8, NCM) have emerged as a research focus for high‐energy‐density battery systems, offering high specific capacity (>200 mAh g^−1^) coupled with reduced cobalt‐related costs [2, 3]. Among these, single‐crystal Ni‐rich NCM cathodes demonstrate substantially enhanced electrode compaction density during manufacturing due to their integral crystal structure, large particle size, and ultralow grain boundary density [4, 5, 6]. This characteristic directly translates to superior volumetric energy density in batteries, a decisive advantage for space‐constrained applications such as electric vehicles and portable electronics [7, 8, 9]. Furthermore, single‐crystal Ni‐rich NCMs exhibit superior mechanical/chemical robustness compared to their polycrystalline counterparts, positioning them as pivotal candidates for next‐generation high‐energy Ni‐rich cathodes [10, 11].

However, synthesizing single‐crystal NCM cathodes requires sintering temperatures substantially higher than those for polycrystalline analogues, aggravating lithium volatilization [12, 13, 14, 15, 16]. Volatile lithium species irreversibly react with ambient CO_2_/H_2_O, forming RLCs dominated by Li_2_CO_3_/LiOH [17, 18]. Concurrently, precursor formulations demand ultrahigh lithium‐to‐transition‐metal ratios (Li/(Ni+Co+Mn) >1.05) to compensate for lithium loss during high‐temperature processing, further exacerbating RLCs accumulation [19, 20]. Consequently, single‐crystal NCM systems face significantly more severe RLCs challenges than polycrystalline counterparts. These residual species not only degrade electrochemical performance and safety but also induce gelation of electrode slurries, thereby hampering scalable battery manufacturing [21, 22, 23]. Furthermore, RLCs trigger parasitic interfacial side reactions, substantially increasing electrode/electrolyte interfacial impedance and ultimately culminating in rapid rate‐capability fading—a critical limitation for fast‐charging single‐crystal cathodes [24, 25, 26].

Substantial research efforts have been dedicated to reducing RLCs in Ni‐rich NCM cathodes, a critical approach for mitigating interfacial impurities' detrimental effects on fast‐charging performance. Primary modification strategies include surface coating [27, 28, 29, 30, 31], lattice doping [32, 33, 34, 35, 36, 37], and chemical washing (aqueous/acidic/alcoholic treatments) [38, 39, 40]. Ryu [41] developed a cobalt‐containing aqueous washing protocol that simultaneously removes residual lithium and forms protective coatings on Ni‐rich layered cathodes. Jiang [42] leveraged the alkaline nature of RLCs to transform them into functional molecular layers during slurry processing, enabling stable cathode‐electrolyte interphase (CEI) formation. Li [43] exploited the reactivity of RLCs (e.g., LiOH/Li_2_CO_3_) toward samarium during high‐temperature synthesis, consuming RLCs while generating a fast‐ion‐conducting samarium oxide coating.

Aqueous washing, recognized as a simple, efficient, and economical method, has gained widespread industrial adoption [44, 45]. When combined with secondary annealing, this process mitigates particle agglomeration, removes RLCs, and optimizes electrochemical performance. However, prolonged washing induces irreversible Li^+^/H^+^ exchange that severely depletes surface lattice lithium/oxygen [46, 47, 48]. This structural degradation triggers the reduction of surface Ni^3+^ to Ni^2+^, which reconstructs into a rock‐salt phase (Fm‐3m NiO) during annealing. Concurrently, washing‐induced damage to the layered structure elevates interfacial resistance, impairing rate capability [49, 50, 51]. Consequently, directly applying polycrystalline NCM post‐washing strategies to single‐crystal counterparts may extend beyond marginal capacity loss to profound structural degradation and performance deterioration.

This work demonstrates that 3‐min ultrafast aqueous washing of LiNi_0.85_Co_0.10_Mn_0.05_O_2_ single crystals (SC‐NCM85) efficiently removes RLCs while preventing severe surface reconstruction, enabling superior fast‐charging capability and cycling stability.

## 2 Results and Discussion

The SC‐NCM85 was synthesized from a coprecipitation‐derived Ni_0.85_Co_0.10_Mn_0.05_(OH)_2_ precursor (Figure S1) using a LiOH·H_2_O‐Li_2_SO_4_ mixture as lithium source, followed by calcination at 860°C. The resulting powder (SC‐NCM85) was subjected to aqueous washing in deionized water (∼20°C) with magnetic stirring (650 rpm) for X minutes, then annealed at 600°C in a tube furnace under flowing oxygen. The final samples were designated as SC‐XMin (X = 0.5, 3, and 60).

**FIGURE 1:**
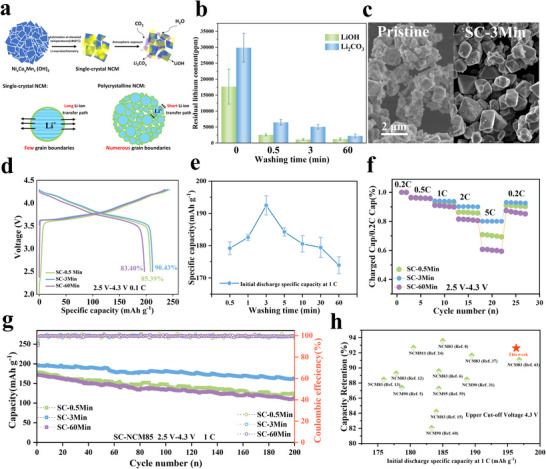
(a) Governing factors and mechanistic origins of rate capability limitations in single‐crystal Ni‐rich layered cathodes. (b) Residual lithium compound (LiOH and Li_2_CO_3_) content (mean ± SD) in SC‐NCM85 cathodes as a function of washing duration; (c) Comparative SEM micrographs of pristine (SC‐Pristine) and 3‐min washed (SC‐3Min) particles; (d) Initial charge/discharge profiles of SC‐0.5Min, SC‐3Min, and SC‐60Min cathodes cycled at 0.1 C (2.5–4.3 V); (e) Initial specific discharge capacities (mean ± SD) measured at 1 C rate for the secondary‐annealed cathodes with controlled washing durations; (f) Normalized discharge capacity retention at varied C‐rates (reference: 0.2 C‐charged capacity); (g) Cycling performance at 1 C (2.5–4.3 V) for SC‐0.5Min, SC‐3Min, and SC‐60Min; (h) Comparative benchmarking of initial 1 C discharge capacity and 100‐cycle reversible capacity retention for this work against reported Ni‐rich cathodes (80%–95% Ni).

As illustrated in Figure 1a, over‐excessed lithium sources and much higher calcination temperature cause serious RLCs on Ni‐rich single crystals than polycrystals, together with a longer Li^+^ diffusion pathway leading to inferior Li^+^ (de)intercalation reversibility [14, 24, 26, 52, 53, 54, 55]. Acid–base titration and ion chromatography analysis (Figure 1b; Table S1) revealed that the initial SC‐NCM85 contained high levels of RLCs: LiOH and Li_2_CO_3_ both exceeded 10 000 ppm, while Li_2_SO_4_ was significantly higher at over 100 000 ppm, as expected. RLCs contents decreased markedly after only 0.5 min of washing, and continued falling along increase of washing time. Morphological evolution captured in Figure 1c reveals pristine crystals (∼2 µm diameter) obscured by solid lithium deposits causing severe agglomeration, while after 3‐min washing well‐defined polyhedral single crystals show clean and smooth surfaces. Critically, Rietveld refinement of XRD patterns (Figure S2, Table S2) shows negligible lattice parameter variations across all samples, confirming that washing‐secondary annealing preserves bulk crystallographic integrity [38, 51, 56].

Although the minimum RLCs is achieved by the SC‐60Min sample, the SC‐3Min sample delivers the largest reversible capacity 212.11 mAh g^−1^ and the highest initial coulombic efficiency (ICE) 90.43% at 0.1 C, while the SC‐60Min sample performs worst, as compared in Figure 1d. Figure 1e compares the reversible capacities at 1 C, revealing that the SC‐3Min sample achieves the highest capacity, and 1 C discharge capacity progressively decreases with extended washing time, demonstrating that industrially adopted prolonged washing (typically hours) is counterproductive.

Following the Department of Energy (DOE) extreme fast charging (XFC) protocol [57, 58], we evaluated fast‐charging performance of SC‐0.5Min, SC‐3Min, and SC‐60Min cathodes under progressively increasing rates (0.2, 0.5, 1, 2, and 5 C), with constant C/3 discharge. As shown in Figure 1f, both over‐washed (SC‐60Min) and under‐washed (SC‐0.5Min) cathodes exhibit significantly reduced charge capacity at elevated rates. Remarkably, SC‐3Min maintains >80% of its 0.2 C‐charged capacity even at 5 C satisfying critical XFC targets. Long‐term cycling stability at 1 C further differentiates performance in Figure 1g. SC‐3Min delivers the highest initial capacity (196.32 mAh g^−1^) and superior retention (162.77 mAh g^−1^ after 200 cycles). SC‐0.5Min and SC‐60Min suffer lower initial capacities (178.5/171.67 mAh g^−1^) and accelerated fade. Figure 1h benchmarks the initial 1 C discharge capacity and 100‐cycle capacity retention of our ultrafast washing treated Ni‐rich single crystals against literature‐reported Ni‐rich layered cathodes (80%–95% Ni) [59, 60]. The literature data reveal a characteristic trade‐off relationship: materials with high initial capacity generally exhibit limited cycle life, while those with superior cycling stability show moderate‐to‐low initial capacity. Notably, our ultrafast washing strategy (highlighted in red star) demonstrates simultaneous improvements in both metrics, outperforming other literature analogues.

Building upon these observations, a systematic comparative investigation of materials processed with varied washing durations is warranted to elucidate the structure‐property evolution mechanisms linking temporal washing parameters to SC‐NCM85 performance, particularly focusing on aqueous processing‐induced alterations in surface structure and their underlying mechanisms.

The effects of aqueous washing time on surface chemical composition and elemental valence states were systematically investigated by X‐ray photoelectron spectroscopy (XPS). Figure S3 presents high‐resolution C 1s and S 2p spectra for SC‐Pristine, SC‐0.5Min, SC‐3Min, and SC‐60Min. Peak deconvolution reveals significant attenuation of the CO_3_^2−^ characteristic peak (289.8 eV) after merely 0.5‐min washing, with further intensity reduction upon prolonged processing. Concurrently, the sulfate signature (Li_2_SO_4_) in S 2p spectra disappears completely post 0.5‐min washing. These semi‐quantitative XPS results align with the quantitative lithium impurity trends in Figure 1b and Table S1.

**FIGURE 2:**
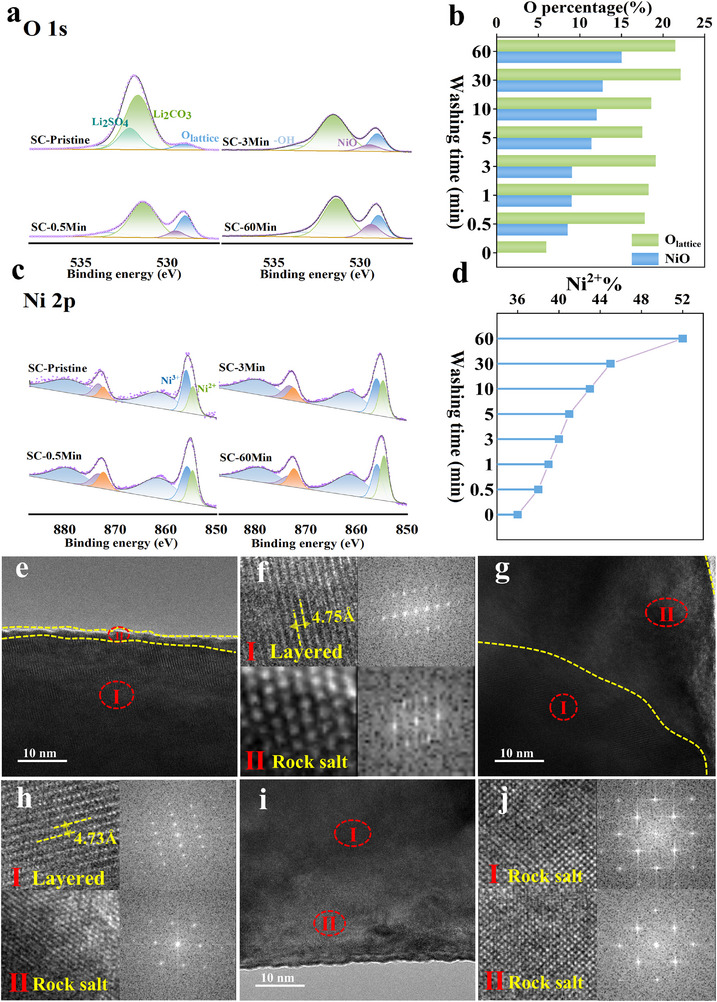
(a) O 1s and (c) Ni 2p XPS spectra with corresponding (b) NiO and (d) Ni^2+^ atomic concentrations for SC samples (SC‐Pristine, SC‐0.5Min, SC‐3Min, SC‐60Min); TEM, HR‐TEM and FFT images for (e,f) SC‐3Min, (g,h) SC‐30Min, and (i,j) SC‐60Min.

Deconvolution of O 1s spectra (Figure 2a) identifies four primary components: 532.2 eV (Li_2_SO_4_), 531.5 eV (Li_2_CO_3_), 530.5 eV (NiO), and 529 eV (lattice oxygen).

The Li_2_SO_4_ component vanishes after 0.5‐min washing. Aqueous processing inevitably triggers Li^+^/H^+^ exchange, causing substantial depletion of surface lattice lithium/oxygen [47, 48]. This structural degradation promotes reduction of surface Ni^3^⁺ to Ni^2+^, subsequently reconstructing into rock‐salt NiO during annealing. This evolution manifests in O 1s spectra as a monotonic increase in NiO component (530.5 eV) relative peak area with extended washing time (Figure 2b; Figure S4) [51]. Further verification through Ni 2p fine spectra analysis (Figure 2c) demonstrates significant variations in the intensity ratio between characteristic peaks at 856 eV (Ni^3+^) and 854.6 eV (Ni^2+^) with progressive washing duration, where the systematic increase in Ni^2+^ proportion quantified from 36% to 52% across the sample series (Figure 2d; Figure S4) exhibits direct correlation with surface rock‐salt NiO formation [38], indicating accelerated degradation of the surface layered structure. Consequently, it can be inferred that the enhanced performance of SC‐3Min in Figure 1 stems from optimized washing: precise control of cathode‐water contact duration enables effective removal of RLCs while minimizing aqueous corrosion.

Although extended washing effectively reduces RLCs content, it concomitantly promotes thicker NiO rock‐salt reconstruction layers that compromise electrochemical performance, as corroborated by high‐resolution transmission electron microscopy (HR‐TEM) characterization of SC‐3Min, SC‐30Min, and SC‐60Min samples (Figure 2e–j). Quantitative analysis reveals that SC‐3Min displays a 2–4 nm disordered rock‐salt layer overlaying the bulk layered phase, with fast Fourier transform (FFT) analysis of Region I (Figure 2f) maintaining discernible superlattice reflections that confirm preserved Li/TM cation ordering [26, 61]. SC‐30Min develops a 20–30 nm thick NiO‐type rock‐salt phase (Fm‐3m space group, Figure 2g,h). While Figure 2i,j shows that SC‐60Min particle surfaces develop thicker rock‐salt reconstruction layers, rendering the underlying layered phase within single crystals nearly undiscernible, this structural evolution accounts for the low ICE observed for SC‐60Min in Figure 1d [47, 48, 49]. The pronounced difference in rock‐salt phase layer thickness observed by HR‐TEM strongly correlates with water‐washing duration within this range, a trend qualitatively consistent with the XPS surface analysis in Figure 2a–d. Prolonged water washing facilitates persistent Li^+^/H^+^ ion exchange, causing severe depletion of surface lattice lithium and oxygen species. This degradation ultimately compromises the structural integrity of the surface layered phase in single‐crystal particles [62, 63, 64].

Typically, irreversible phase transitions to electrochemically inert spinel/rock‐salt phases occur under high‐voltage or prolonged cycling conditions, leading to the degradation of the layered structure into a rock‐salt phase within the surface and near‐surface regions of Ni‐rich NCM cathodes [65, 66]. Studies have demonstrated that the accumulation of the rock‐salt phase not only blocks Li^+^ diffusion pathways, but more critically, the pronounced lattice mismatch between this phase and the internal layered bulk effectively impedes Li ion extraction at high states of charge [66, 67]. This directly contributes to irreversible active lithium loss and significant capacity/voltage fading, as reported by Zhao and Song [50, 68]. Furthermore, the formation of the rock‐salt phase induces intragranular microcracks. The heterogeneous distribution of these cracks severely compromises the mechanical integrity of the material, exacerbating structural degradation [18, 69, 70]. Additionally, the electrochemically inactive rock‐salt phase formed at the electrode surface/interface substantially increases interfacial impedance. This phenomenon not only hinders charge transfer kinetics but also constitutes a key factor in the progressive deterioration of overall electrochemical performance, including rate capability and cycling stability [71, 72, 73]. In summary, the ultrafast water‐washing treatment applied to Ni‐rich cathodes results in a reduced degree of surface reconstruction layer. This characteristic contributes to maintaining favorable kinetics and stability in the SC‐NCM85 cathode.

**FIGURE 3:**
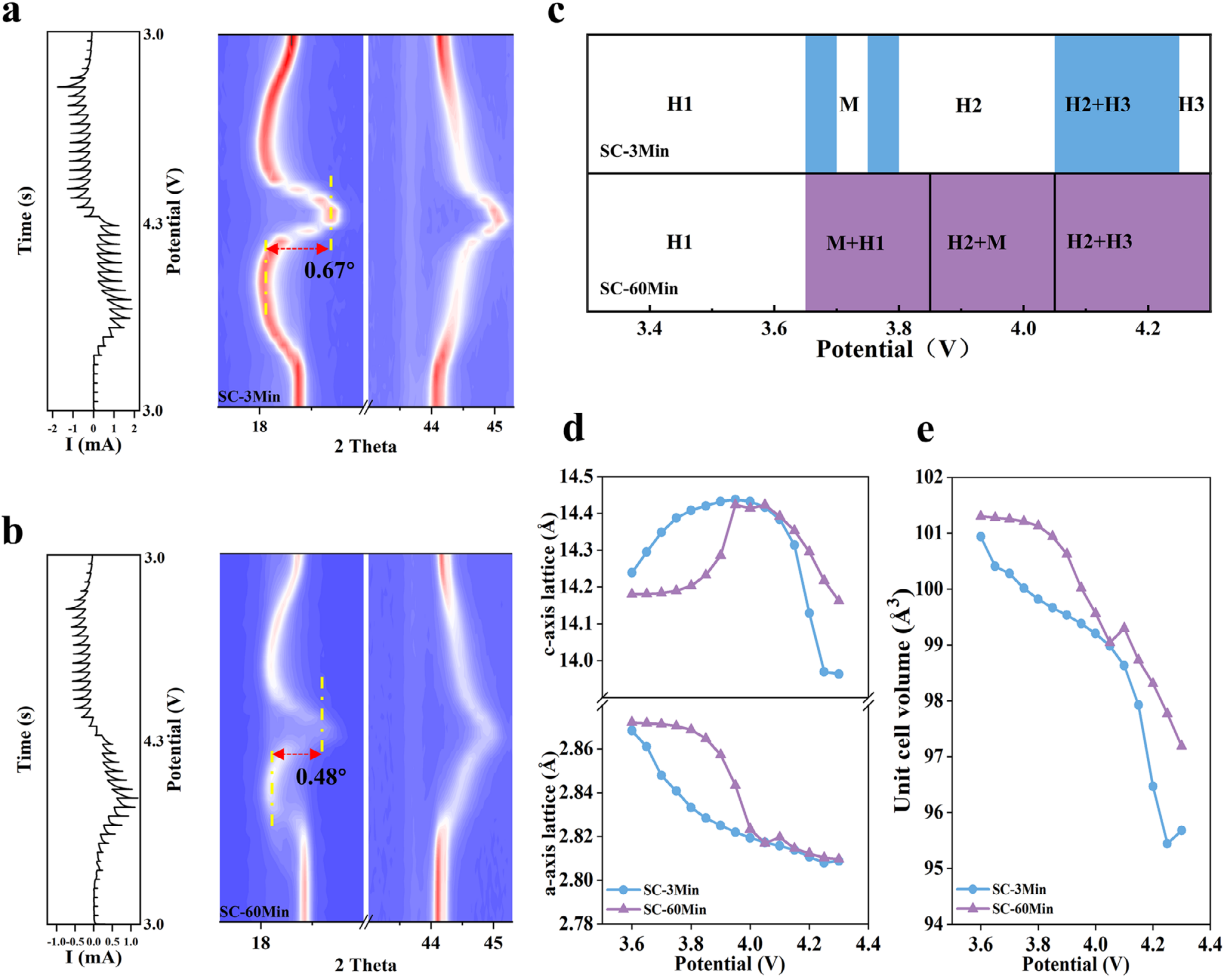
In situ XRD monitoring: (a,b) Potential‐dependent structural evolution of SC‐3Min and SC‐60Min; (c) Phase transformation pathways during delithiation; (d,e) Rietveld‐refined lattice dynamics: (d) *a*‐ axis /*c*‐axis parameters and (e) unit cell volume evolution during cycling.

To elucidate the impact of the rock‐salt surface reconstruction layer (induced by excessive water washing) on the reversibility of structural phase transitions across varying states of charge (SOC), constant‐potential intermittent titration technique (PITT) coupled with in situ XRD monitoring was performed on both SC‐3Min and SC‐60Min cathodes [74]. Figure 3a,b presents the corresponding XRD contour plots, depicting the evolution of diffraction patterns under applied potential. During the H1→M→H2 phase transition sequence, the (003) diffraction peak shifts toward lower angles for both cathodes. This shift corresponds to lattice expansion along the *c*‐axis, arising from enhanced electrostatic repulsion between oxygen layers upon Li^+^ extraction from the lithium slab [75]. As the potential further increases, the (003) peak subsequently shifts back toward higher angles, indicating rapid contraction of the interlayer spacing and pronounced *c*‐axis compression. During discharge (potential decrease), the phase transformations for both cathodes follow the reverse pathway. Notably, distinct differences in phase transition behavior are observed: The (003) peak of SC‐3Min shifts by 0.67° toward higher angles during the H2→H3 transition, significantly greater than the 0.48° shift observed for SC‐60Min. Furthermore, SC‐60Min exhibits systematic phase transition hysteresis, manifested as kinetically retarded phase evolution. Retention of the H1 phase generates significantly blurred 003 diffraction contours throughout the H1→M→H2 transition in Figure 3b. The corresponding peak splitting observed in Figure S5 corroborates multiphase coexistence [67]. Concurrently, H2/H3 phase coexistence emerges in SC‐60Min at high SOC, inducing analogous (003) peak anomalies and signifying heterogeneous phase transformation. In contrast, SC‐3Min demonstrates continuous and smooth evolution of the (003) peak, indicative of a predominantly solid‐solution (single‐phase) reaction mechanism. Figure 3c summarizes the phase evolution pathways for both cathodes, derived from detailed analysis of the in situ XRD data during charge/discharge, corroborating the above observations. Compared to SC‐60Min, SC‐3Min traverses a narrower two‐phase region during charging. Conversely, SC‐60Min undergoes a sluggish and heterogeneous phase transformation process, characterized by extended two‐phase coexistence throughout the entire transition.

Quantitative analysis of lattice parameters via XRD refinement during the charging process reveals distinct differences (Figure 3d,e). SC‐60Min exhibits a maximum *c*‐axis contraction magnitude of 1.82%, significantly lower than the 3.28% observed for SC‐3Min. These quantitative findings demonstrate that the rock‐salt phase on the SC‐60Min single‐crystal surface significantly suppresses lattice expansion/contraction during charging. This suppression originates from the phase's intrinsically low Li^+^ conductivity and its pinning effect on the bulk layered structure, which collectively restrict lithium‐ion deintercalation‐induced structural changes [61, 66, 67, 76, 77]. In the low SOC regime, the reconstructed surface rock‐salt layer of a certain thickness further exacerbates the intrinsically sluggish redox kinetics of the Ni‐rich single crystal, thereby inducing retarded phase‐transition behavior [76, 78]. Furthermore, although redox kinetics accelerate with increasing SOC [79, 80, 81], the thick rock‐salt layer in SC‐60Min induces significant interfacial lattice strain between itself and the bulk layered structure at higher SOCs. This strain results in a pinning effect, where the rock‐salt phase restricts the contraction of the adjacent layered lattice planes along the *c*‐axis. Consequently, this impedes the further lattice contraction required for continued delithiation within the layered structure [66], similarly contributing to sluggish phase transition behavior in SC‐60Min. Collectively, these findings demonstrate that excessive water washing induces detrimental surface reconstruction on single‐crystal materials, significantly hindering their phase transition dynamics and profoundly compromising the electrochemical performance of the cathode.

**FIGURE 4:**
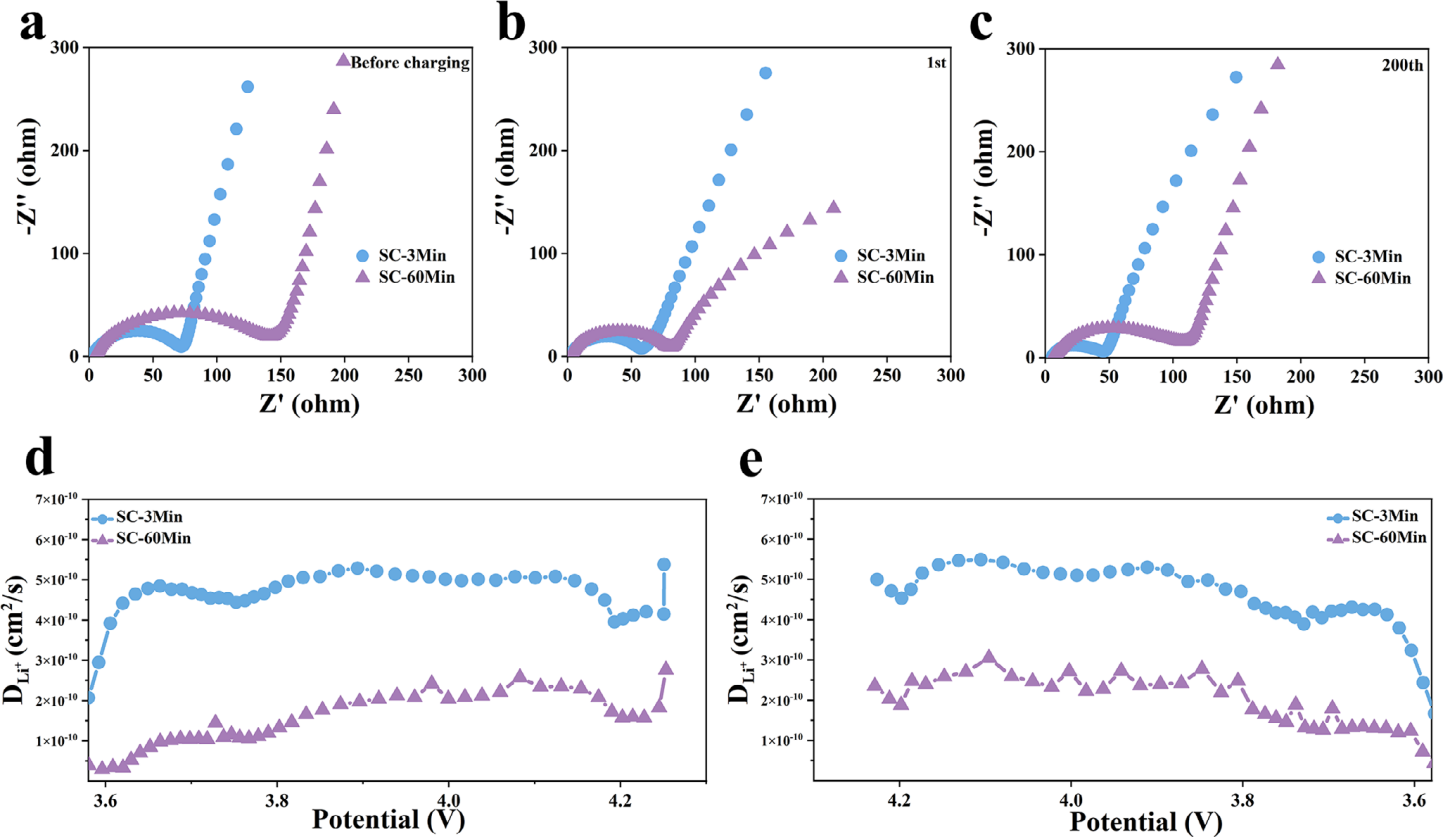
Conductivity and Li^+^ diffusivity evolution: (a–c) EIS spectra of SC‐3Min and SC‐60Min (a) before charging, (b) after 1 cycle, (c) after 200 cycles; (d,e) GITT‐derived Li^+^ diffusion coefficients during (d) charge and (e) discharge processes.

Ionic conductivity critically governs fast‐charging performance. To elucidate washing duration effects on impedance evolution, electrochemical impedance spectroscopy (EIS) was performed on SC‐3Min and SC‐60Min cathodes [37, 80, 81]. Figure 4a–c present the Nyquist plots of coin‐type half‐cells at different stages: before cycling, after 1 cycle, and after 200 cycles. Each plot consists of a high‐frequency semicircle, corresponding to the charge transfer resistance (Rct), and a low‐frequency linear region, which represents the Warburg impedance (Zw) associated with Li⁺ diffusion [82]. The corresponding equivalent circuit is shown in Figure S6. The electrochemical impedance fitting results of SC‐3Min and SC‐3Min are shown in Table S3. Pre‐cycling Rct values were 69.1 Ω for SC‐3Min vs. 131.2 Ω for SC‐60Min (Figure 4a). After the 1 stand 200 cycles, Rct increased to 69.9 and 97.1 Ω for SC‐60Min (Figure 4b,c), while SC‐3Min exhibited lower values of 52.3 and 37.2 Ω, respectively, demonstrating superior interfacial stability during prolonged cycling. Galvanostatic intermittent titration technique (GITT) measurements further revealed higher Li^+^ diffusion coefficients (D_Li+_) for SC‐3Min vs. SC‐60Min during charge/discharge (Figure 4d,e). This confirms that ultrafast washing effectively removes RLCs without impeding interfacial Li^+^ transport, thereby enhancing diffusion kinetics. In contrast, the thick NiO‐type rock‐salt reconstruction layer on SC‐60Min particles increases polarization and impedance, exhibiting a much higher effective energy barrier for Li^+^ diffusion than the layered phase [51]. This disordered phase exacerbates kinetic limitations and induces heterogeneous phase transitions during electrochemical processes.

**FIGURE 5:**
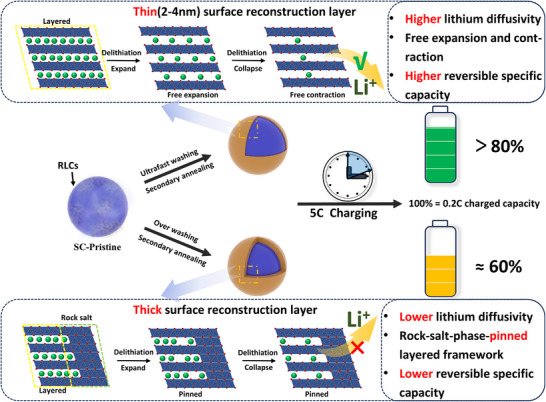
Comparative schematic illustrating the effects of ultrafast washing vs. over‐washing coupled with secondary annealing on structural evolution, lithium diffusion kinetics, and electrochemical performance in SC‐NCM85 cathode materials during cycling.

The impact of washing and annealing protocols on the electrochemical performances of SC‐NCM85 cathodes is summarized in Figure 5. Ultrafast washing effectively eliminates RLCs from single‐crystal surfaces, substantially enhancing interfacial stability and fast‐charging capability. However, over‐washing (>30 min) induces severe Li^+^/H^+^ exchange, triggering the formation of a NiO‐like rock‐salt reconstruction layer with substantial thickness during subsequent annealing. This reconstructed phase exhibits intrinsically low lithium diffusivity and exerts a strong pinning effect on the layered bulk framework during electrochemical cycling, thereby constraining the lattice contraction/expansion essential for reversible (de)intercalation. Such rock‐salt phase reconstruction initiates structural fatigue and degradation in single‐crystal particles, manifesting as intrinsically low reversible capacity from initial cycles and significantly compromised rate performance compared to ultrafast‐washed counterparts.

## 3 Conclusion

This study demonstrates that aqueous washing exerts dual influences on electrode interfacial stability. Efficient removal of RLCs is achieved through moderate, brief washing, thereby concurrently mitigating the exacerbation of surface reconstruction. Conversely, excessive water washing indirectly triggers catastrophic reconstruction of the cathode surface structure, forming a significantly thick and irreversible disordered rock‐salt phase layer. Within this rock‐salt phase, the electrochemically inactive NiO component exhibits a higher lithium‐ion diffusion energy barrier compared to the layered NCM phase, transforming it into a barrier for both Li ion and electron transport. Consequently, this leads to a significant deterioration in the reversible capacity of single‐crystal NCM materials under high‐rate conditions. Notably, when the detrimental surface reconstruction layer induced by excessive washing reaches a critical thickness, it prematurely triggers fatigue failure behavior typically observed only in the late stages of long‐term cycling for single‐crystal Ni‐rich cathodes, severely compromising the material's structural integrity.

In summary, this work applies a well‐controlled ultrafast protocol to effectively mitigate surface RLCs, synthesis byproducts in single‐crystal Ni‐rich cathodes, while reducing the risks of aqueous washing‐induced structural degradation. By replacing conventional prolonged washing with this approach, processing is streamlined and post‐treatment energy consumption is reduced. Crucially, this post‐treatment protocol achieves interfacial optimization through RLCs suppression and preserves surface layered structural integrity via limited surface reconstruction degradation, thereby contributing to enhanced fast‐charging capability in these cathodes. These advances establish practically viable processing parameters for high‐performance single‐crystal Ni‐rich layered oxide cathodes.

## Conflicts of Interest

The authors declare no conflicts of interest.

## Supporting information




**Supporting File**: advs73433‐sup‐0001‐SuppMat.docx.

## Data Availability

The data that support the findings of this study are available from the corresponding author upon reasonable request.
