# Supplementary material for: Achieving Fast Charging and Superior Cycling Stability Single‐Crystal Ni‐Rich Cathodes by Ultrafast Aqueous Washing
Source: Adv Sci (Weinh). 2025 Dec 23;13(10):e17421. doi: 10.1002/advs.202517421 (PMC12915119; doi:10.1002/advs.202517421)
Supplement: Supplementary file 1 — Supporting File: advs73433‐sup‐0001‐SuppMat.docx. [file ADVS-13-e17421-s001.docx]

**Supporting Information**

for

**Achieving Fast Charging and Superior Cycling Stability Single-Crystal Ni-Rich Cathodes by Ultrafast Aqueous Washing**

Kaixin Liu^a^, Jia Yang^a^, Huaping Wang^a,b^, Yongtao Tan^a^, Dongdong Fan^a,b^, Yongjian Cui^a,b^, YunJian Liu^c^, Xiaoyan Li^d^, Hailong Wang^a^*^,b^

^a^School of Materials and New Energy, Ningxia University, Yinchuan 750021, China

^b^Helanshan Laboratory, Yinchuan 750021, China

^c^School of Materials Science & Engineering, Jiangsu University, Zhenjiang 212013, China

^d^Ningxia Hanyao Rich Lithium Technology Co.,Ltd., Yinchuan 750021, China

Corresponding Author E-mail Address: wanghailong@nxu.edu.cn;

Keywords: Ni-rich oxides, single crystal, fast charging capability, cycling stability

**Experimental Section**

**Materials synthesis:** The Ni_0.85_Co_0.10_Mn_0.05_(OH)_2_ precursor was synthesized via the coprecipitation method. Employing a nitrogen-blanketed continuous stirring reactor, the precipitation reaction was conducted with a base liquid at pH 11.60, 0.5 mol·L^-1^ ammonia, and a temperature of 50 °C. To obtain the precipitate, a 2 mol·L^-1^ solution of the metal salts NiSO_4_·6H_2_O, CoSO_4_·7H_2_O, and MnSO_4_·H_2_O was prepared in stoichiometric proportions and dissolved, followed by the addition of sodium hydroxide solution and ammonia solution into the reactor maintained at a pH of 11.6. The obtained precursor was subjected to filtration and multiple washes, followed by drying in an oven at 100 °C for 20 hours. The Ni_0.85_Co_0.10_Mn_0.05_(OH)_2_ precursor was mixed with LiOH·H_2_O/Li_2_SO_4_ in a ratio of 3:1 and ground with a molar ratio of Li/(Ni + Co + Mn) = 1.5:1. The mixture was presintered at 460 °C for 6 hours, followed by calcination at 860°C for 12 hours under an O_2_ atmosphere with a heating rate of 5 °C min^-1^. The obtained LiNi_0.85_Co_0.10_Mn_0.05_O_2_ (NCM85) powder was washed with deionized water at ~20 ℃ (weight ratio of NCM85 to H_2_O = 1:30) under continuous stirring at 650 rpm for durations of 0.5 min, 3 min and 60 min to remove surface residual lithium. The powder was then collected by vacuum filtration, rinsed with ethanol, and dried at 80 ℃ for 10 min under vacuum to remove residual solvent. Subsequently, the powder was annealed at 600 ℃ under an O_2_ atmosphere for 6 hours. The resulting materials were labeled SC-XMin (X= 0.5, 3, 60).

**Material Characterization:** X-ray diffraction (XRD) data were collected using the step mode (0.01° per step) on a 9 kW Rigaku SmartLab diffractometer (Rigaku, Japan). Rietveld refinement was carried out to extract structural information using GSAS Ⅱ software. The morphology of particles was characterized by scanning electron microscopy (SEM) using a Hitachi SU5000 microscope (Hitachi, Japan). The particle microstructure was evaluated by high-resolution transmission electron microscopy (HR-TEM) using a JEOL JEM-F200 microscope (JEOL, Japan). X-ray photoelectron spectroscopy (XPS) data were collected using a Thermo Scientific ESCALAB 250XI spectrometer (Thermo Fisher Scientific, USA). Ion chromatography (IC) analyses were performed using a Dionex ICS5000+ system (Thermo Fisher Scientific, USA).

The residual lithium content in each sample was determined by acid-base titration. The titration was performed using 0.1 mol·L^-1^ hydrochloric acid (HCl) as the titrant. A mixed indicator of methyl red and bromothymol blue (both at 1 mol·L^-1^), as well as a colorless phenolphthalein solution (1 mol·L^-1^), were employed. Initially, a few drops of the colorless phenolphthalein indicator were added, turning the solution red. Titration was stopped when the red color completely disappeared, marking the first equivalence point (corresponding to the neutralization of strong base, e.g., LiOH). The volume of HCl consumed at this stage was recorded as V_1_. Subsequently, the mixed methyl red-bromothymol blue indicator was introduced, resulting in a dark green solution. Titration was continued until the solution turned dark red, marking the second equivalence point (corresponding to the neutralization of weak base, e.g., Li_2_CO_3_). The additional volume of HCl used in this stage was recorded as V_2_. The content of surface residual lithium compounds was then calculated based on V_1_ and V_2_ using the following formula:

$$\mathbf{LiOH}\left( \boldsymbol{wt.\%} \right)\boldsymbol{=}\left[ \frac{\left( \boldsymbol{2}\boldsymbol{V}_{\boldsymbol{1}}\boldsymbol{-}\boldsymbol{V}_{\boldsymbol{2}} \right)\boldsymbol{\times}\boldsymbol{C}_{\boldsymbol{HCl}}\boldsymbol{\times}\boldsymbol{M}_{\boldsymbol{LiOH}}}{\boldsymbol{1000\times W}} \right]\boldsymbol{\times}\left( \frac{\boldsymbol{A}_{\boldsymbol{1}}}{\boldsymbol{A}_{\boldsymbol{2}}} \right)\boldsymbol{\times100}\left( \boldsymbol{wt.\%} \right)$$

$$\mathbf{L}\mathbf{i}_{\mathbf{2}}\mathbf{C}\mathbf{O}_{\mathbf{3}}\left( \boldsymbol{wt.\%} \right)\boldsymbol{=}\left[ \frac{\left( \boldsymbol{V}_{\boldsymbol{2}}\boldsymbol{-}\boldsymbol{V}_{\boldsymbol{1}} \right)\boldsymbol{\times}\boldsymbol{C}_{\boldsymbol{HCl}}\boldsymbol{\times}\boldsymbol{M}_{\boldsymbol{Li}\boldsymbol{2}\boldsymbol{CO}\boldsymbol{3}}}{\boldsymbol{1000\times W}} \right]\boldsymbol{\times}\left( \frac{\boldsymbol{A}_{\boldsymbol{1}}}{\boldsymbol{A}_{\boldsymbol{2}}} \right)\boldsymbol{\times100}\text{(}\boldsymbol{wt.\%}\text{)}$$

**Electrochemical measurements:** CR2032 coin cells were assembled with Li metal as the counter/reference electrode. The cathode slurry was prepared by mixing active material, Super P, and PVDF (8:1:1 wt%) in an agate mortar, then coated onto Al foil. Electrodes were vacuum-dried with an active material loading of ~5 mg cm^-2^. The electrolyte was 1.2 mol·L^-1^ LiPF_6_ in EC/EMC (3:7 v/v) with 2% VC additive. Half-cells were cycled between 2.5-4.3 V (vs. Li^+^/Li) at 180 mA g^-1^ (1 C = 180 mA g^-1^). Galvanostatic charge-discharge measurements were performed using a NEWARE Battery Test System (Model CT-4008Q-5V50mA-HWX, China). Potential intermittent titration measurements were performed using an electrochemical workstation (Ivium, Netherlands) in coin cells equipped with Be windows for in-situ X-ray diffraction. A 50 mV potential step was applied at each titration point and held for 300 s, followed by XRD acquisition to monitor structural evolution. GITT and EIS measurements were performed on an Ivium Vertex electrochemical workstation (Ivium Technologies, Netherlands).

**Statistical Analysis:** In this study, the initial discharge capacity (Figure 1b) and surface residual lithium content (Figure. 1e) are reported as mean ± standard deviation. The electrochemical performance data (initial discharge capacity) were obtained with a sample size of n = 6, corresponding to six independently fabricated and tested coin cells under each experimental condition, using a Neware battery testing system. Surface residual lithium content was determined by acid–base titration, with a sample size of n = 5 per group, representing five independently synthesized and characterized batches of cathode material; the contents of Li_2_CO_3_ and LiOH were calculated from the titration results. No data transformation or normalization was applied during preprocessing. Data organization and statistical analysis were performed using Origin 2021 (OriginLab Corporation).


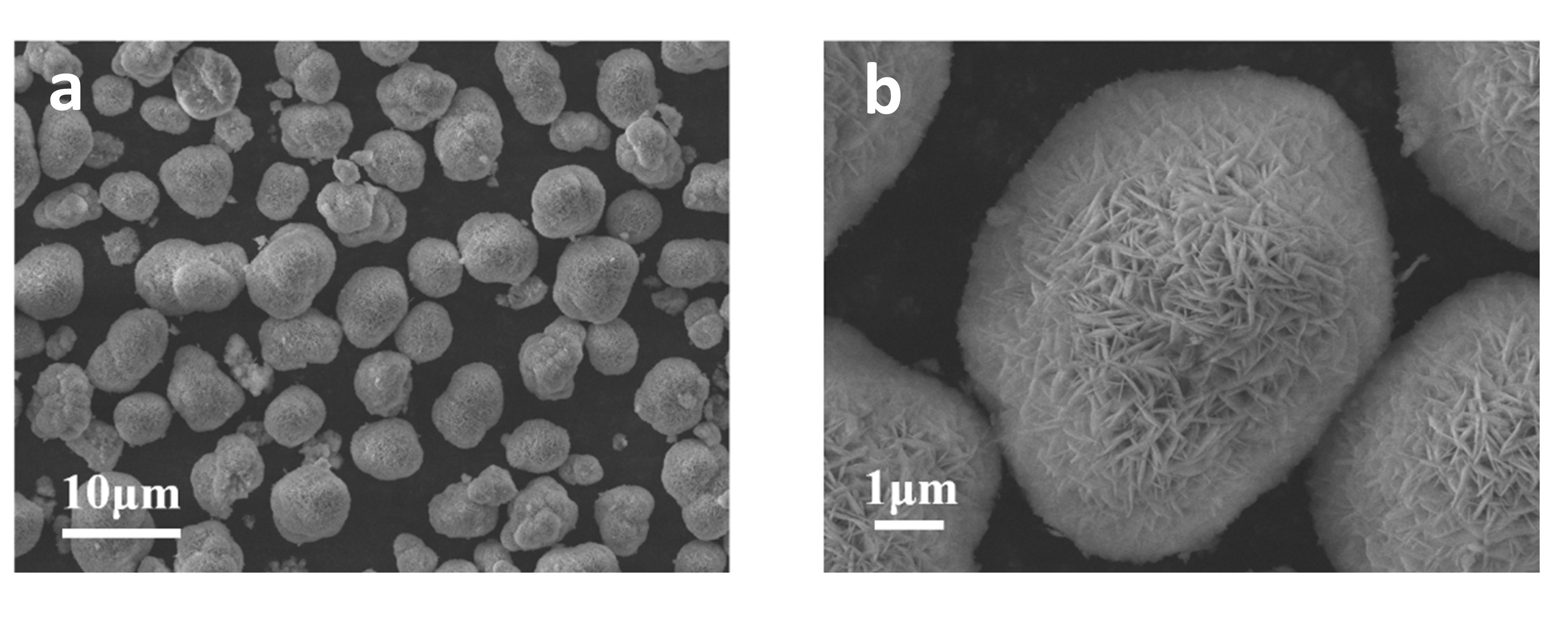


**Figure S1.** SEM observed morphology of Ni_0.85_Co_0.10_Mn_0.05_(OH)_2_ particles: a) individual particle showing densely packed structure, and b) particle sizes are uniformly distributed betwen 6-10 μm.

**
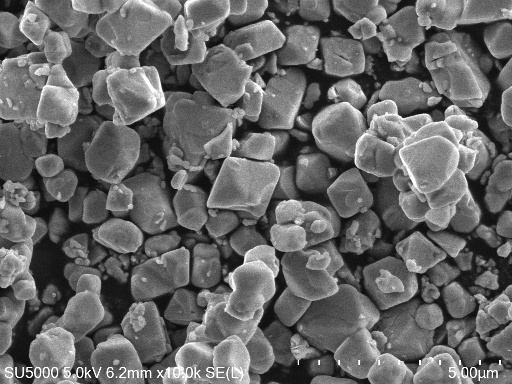

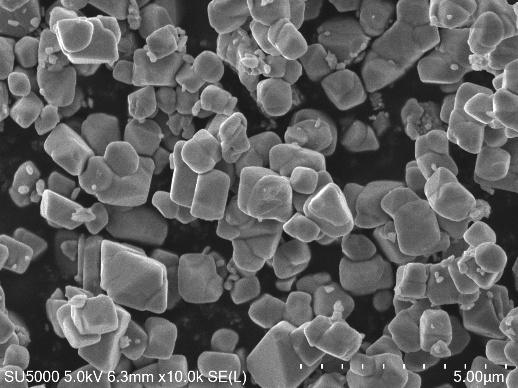
**

**
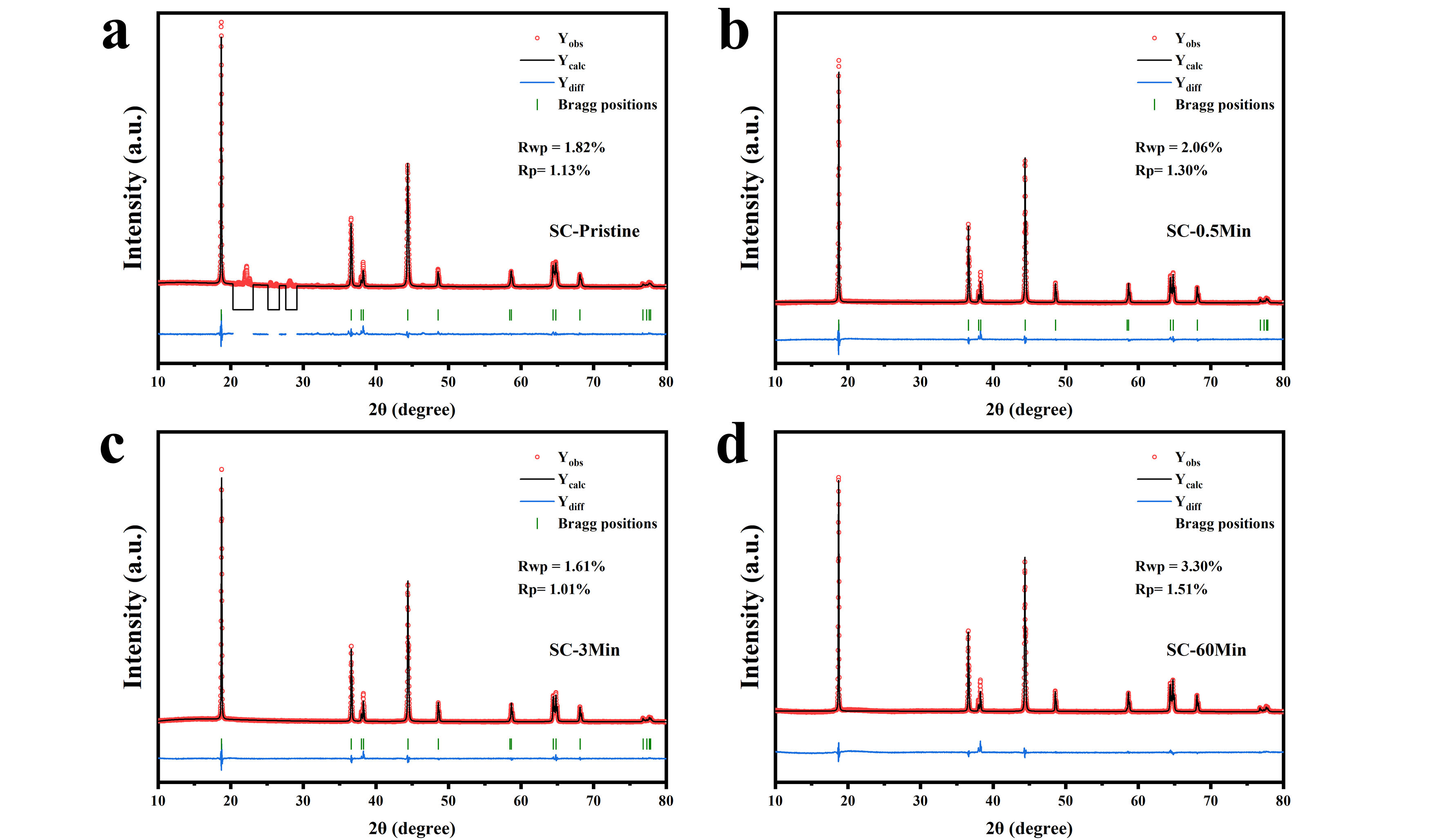
**

**Figure S2.** XRD refinement Rietveld refinement of a) SC-Pristine, b) SC-0.5Min, c) SC-3Min, d) SC-60Min.

**
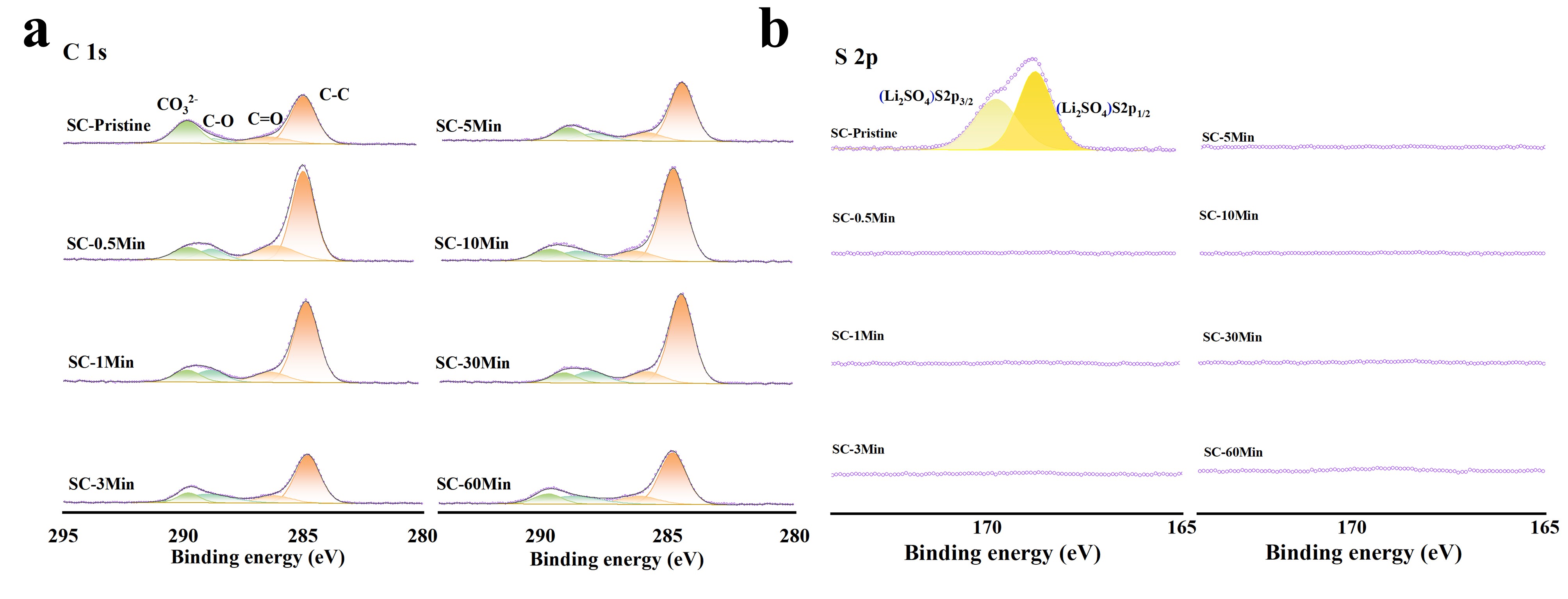
**

**Figure S3.** C 1s a) and S 2p b) from the surface of SC-Pristine, SC-0.5Min, SC-1Min, SC-3Min, SC-5Min, SC-10Min, SC-30Min and SC-60Min.

**
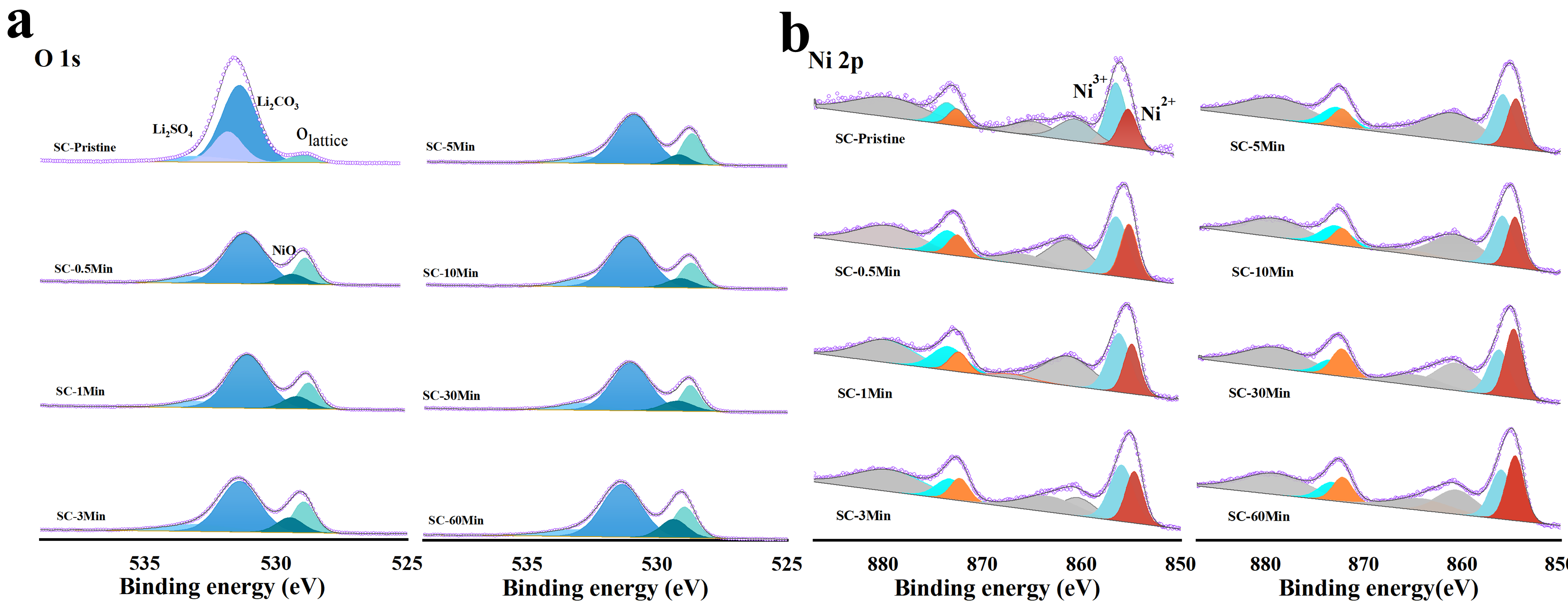
**

**Figure S4.** O 1s a) and Ni 2p b) from the surface of SC-Pristine, SC-0.5Min, SC-1Min, SC-3Min, SC-5Min, SC-10Min, SC-30Min and SC-60Min.


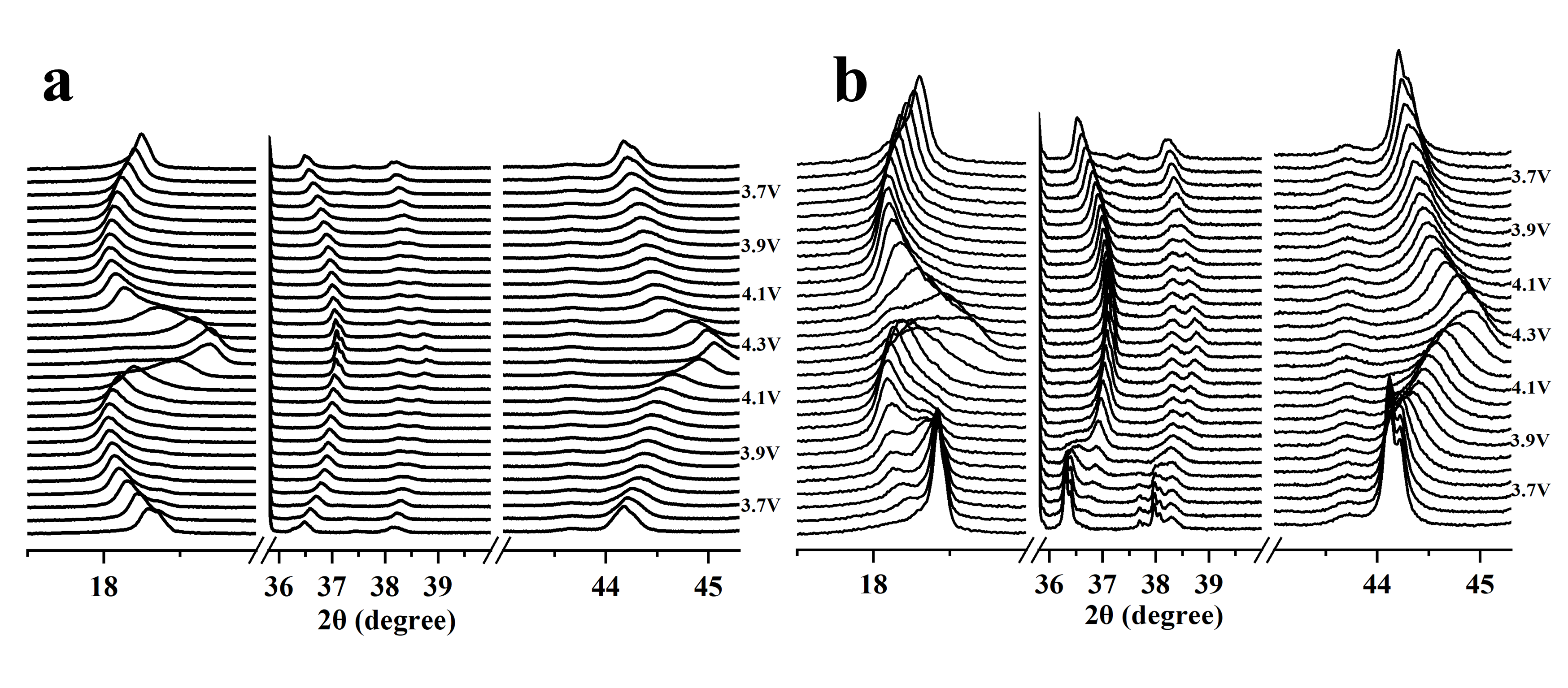


**Figure S5.** Evolution of in-situ XRD diffraction peaks for the a) SC-3Min and b) SC-60Min during electrochemical cycling.


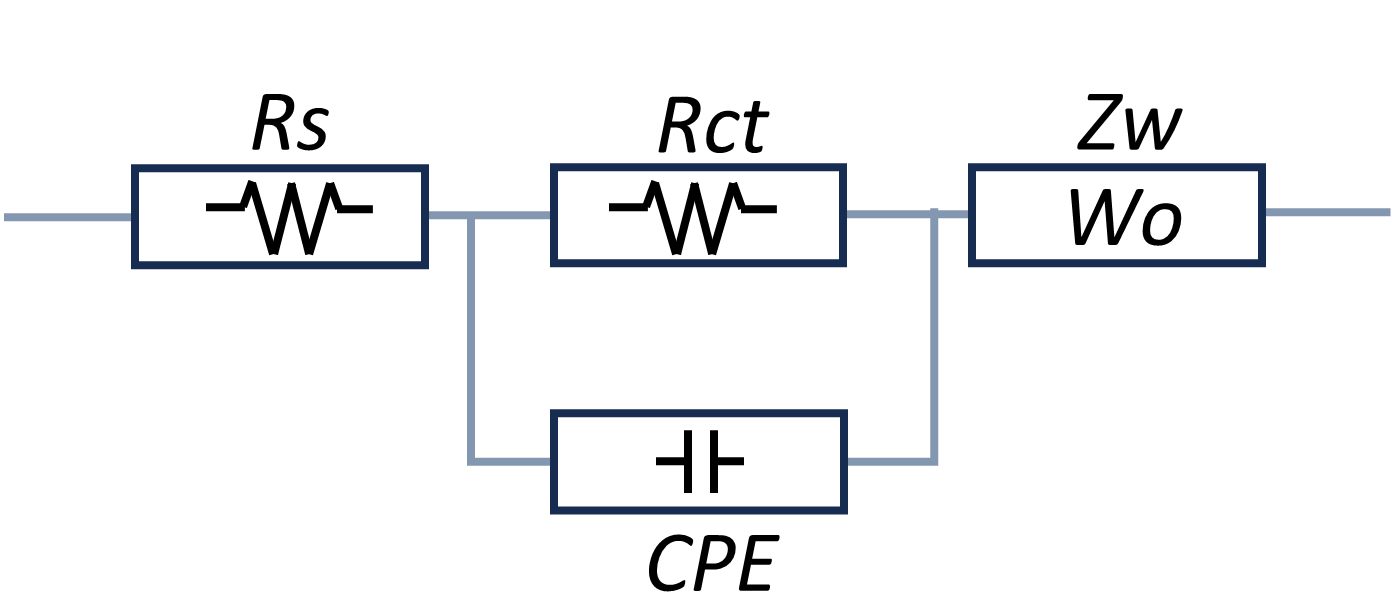


**Figure S6.** Simulated equivalent circuit based on the EIS measurements of SC-3Min and SC-60Min

**Table S1.** Average residual lithium content of the samples.

| **Materials** | **Li_2_CO_3_ (ppm)** | **LiOH (ppm)** | **Li_2_SO_4_ (ppm)** | **Total Li (ppm)** |
| --- | --- | --- | --- | --- |
| **Pristine** | **29349.108** | **19811.701** | **130230** | **29946.18393** |
| **SC-0.5Min** | **6493.4532** | **2461.8544** | **219.954** | **2571.232879** |
| **SC-3Min** | **4533.8904** | **1114.53992** | **52.222** | **1607.324655** |
| **SC-60Min** | **2128.032** | **1379.4048** | **11.272** | **978.7158502** |

$$\text{Total}\text{ }\text{L}\text{i}\mathbf{=}\frac{\boldsymbol{2}\boldsymbol{\times}\text{Atomic}\text{ }\text{weight}\text{ }\text{of}\text{ }\text{Li}}{{\text{molecular}\text{ }\text{weight}\text{ }\text{of}\text{ }\text{Li}}_{\boldsymbol{2}}\text{CO}_{\boldsymbol{3}}}\boldsymbol{\times}\text{Li}_{\boldsymbol{2}}\text{CO}_{\boldsymbol{3}}\mathbf{+}\frac{\text{Atomic}\text{ }\text{weight}\text{ }\text{of}\text{ }\text{Li}}{\text{molecular}\text{ }\text{weight}\text{ }\text{of}\text{ }\text{LiOH}}\boldsymbol{\times}\text{LiOH}\mathbf{+}\frac{\boldsymbol{2}\boldsymbol{\times}\text{Atomic}\text{ }\text{weight}\text{ }\text{of}\text{ }\text{Li}}{{\text{molecular}\text{ }\text{weight}\text{ }\text{of}\text{ }\text{Li}}_{\boldsymbol{2}}\text{SO}_{\boldsymbol{4}}}\boldsymbol{\times}\text{Li}_{\boldsymbol{2}}\text{SO}_{\boldsymbol{4}}$$

**Table S2.** Structural information of pristine SC-Pristine, SC-0.5Min, SC-3Min, SC-60Min.

|  | **Lattice**  **Constant** | | **V[Å^3^]** | **c/a** | **I_(003)_/I_(104)_** | **Rwp** | **Rp** |
| --- | --- | --- | --- | --- | --- | --- | --- |
|  | **a[Å]** | **c[Å]** |  |  |  |  |  |
| **SC-Pristine** | **2.8731** | **14.191** | **101.451** | **4.9391** | **2.15** | **1.82** | **1.13** |
| **SC-0.5Min** | **2.8743** | **14.192** | **101.537** | **4.9374** | **1.32** | **2.06** | **1.30** |
| **SC-3Min** | **2.8718** | **14.192** | **101.367** | **4.9418** | **1.82** | **1.61** | **1.01** |
| **SC-60Min** | **2.8737** | **14.193** | **101.509** | **4.9390** | **1.56** | **2.30** | **1.51** |

**T****able S3.** The values of Rs and Rct of SC-3Min and SC-60Min.

| **Samples** | **Cycling** | **Rs (Ω)** | **Rct (Ω)** |
| --- | --- | --- | --- |
| **SC-3Min** | **before charging** | **1.61** | **69.1** |
|  | **1st** | **1.72** | **52.3** |
|  | **200th** | **4.52** | **37.2** |
| **SC-60Min** | **before charging** | **4.26** | **131.2** |
|  | **1st** | **1.78** | **69.9** |
|  | **200th** | **5.93** | **97.1** |
